# The Plant Ribosome-Inactivating Proteins Play Important Roles in Defense against Pathogens and Insect Pest Attacks

**DOI:** 10.3389/fpls.2018.00146

**Published:** 2018-02-09

**Authors:** Feng Zhu, Yang-Kai Zhou, Zhao-Lin Ji, Xiao-Ren Chen

**Affiliations:** College of Horticulture and Plant Protection, Yangzhou University, Yangzhou, China

**Keywords:** antibacterial, antifungal, antiviral, defense, ribosome-inactivating proteins, systemic resistance

## Abstract

Ribosome-inactivating proteins (RIPs) are toxic *N*-glycosidases that depurinate eukaryotic and prokaryotic rRNAs, thereby arresting protein synthesis during translation. RIPs are widely found in various plant species and within different tissues. It is demonstrated *in vitro* and in transgenic plants that RIPs have been connected to defense by antifungal, antibacterial, antiviral, and insecticidal activities. However, the mechanism of these effects is still not completely clear. There are a number of reviews of RIPs. However, there are no reviews on the biological functions of RIPs in defense against pathogens and insect pests. Therefore, in this report, we focused on the effect of RIPs from plants in defense against pathogens and insect pest attacks. First, we summarize the three different types of RIPs based on their physical properties. RIPs are generally distributed in plants. Then, we discuss the distribution of RIPs that are found in various plant species and in fungi, bacteria, algae, and animals. Various RIPs have shown unique bioactive properties including antibacterial, antifungal, antiviral, and insecticidal activity. Finally, we divided the discussion into the biological roles of RIPs in defense against bacteria, fungi, viruses, and insects. This review is focused on the role of plant RIPs in defense against bacteria, fungi, viruses, and insect attacks. The role of plant RIPs in defense against pathogens and insects is being comprehended currently. Future study utilizing transgenic technology approaches to study the mechanisms of RIPs will undoubtedly generate a better comprehending of the role of plant RIPs in defense against pathogens and insects. Discovering additional crosstalk mechanisms between RIPs and phytohormones or reactive oxygen species (ROS) against pathogen and insect infections will be a significant subject in the field of biotic stress study. These studies are helpful in revealing significance of genetic control that can be beneficial to engineer crops tolerance to biotic stress.

## Introduction

Agricultural crops often suffer from fungi, viruses, and bacteria attacks which negatively cause the survival, biomass production and yield and quality of produces throughout the world (Culbreath et al., 2003; Rodoni, 2009). We have known that plants own some specific metabolic pathways to synthesize lots of valuable proteins, and these proteins can be used for prevention and treatment of diseases (Calixto, 2000). Such as, ribosome-inactivating proteins (RIPs) from plants have been suggested to confer disease resistance (Huang et al., 2008). The RIPs could catalytically inactivate eukaryotic ribosomes which inhibit protein synthesis at translation (de Virgilio et al., 2010).

Various RIPs have been proved to exhibit different of antimicrobial activities, for instance antitumor, antibacterial activity, antifungal activity, and broad-spectrum antiviral activity (Mock et al., 1996; Wang and Tumer, 2000; Stirpe, 2004; Puri et al., 2009; Bian et al., 2010). In agriculture, It is demonstrated *in vitro* and in transgenic plants that RIPs have been connected to defense by antifungal, antibacterial, antiviral and insecticidal activities (Stevens et al., 1981; Wang and Tumer, 2000; Choudhary N.L. et al., 2008; Akkouh et al., 2015). These studies have conferred a wide knowledge base for comprehending the medicinal and biochemical properties of RIPs. Nevertheless, the biological functions of plant RIPs are rarely investigated. Lots of reviews on RIPs have been reported (Nielsen and Boston, 2001; Girbés et al., 2004; Hartley and Lord, 2004b; Stirpe, 2004;Van Damme et al., 2008; de Virgilio et al., 2010; Lord and Hartley, 2010; Ng et al., 2010; Stirpe and Lappi, 2014; Schrot et al., 2015). For example, Bolognesi et al. (2016) provided a historical overview of the biological role of RIPs. However, there are rarely reviews on the biological functions of RIPs in defense against attacks by pathogens (bacteria, fungi, and viruses) and insects. Therefore, we focus on recent study advances, distribution, physiological roles of RIPs and their roles in defense against pathogen and insect attacks.

### Types of Ribosome-Inactivating Proteins

RIPs from plants have been divided into three main types based on their physical properties, including type I, type II, and type III (Olsnes and Pihl, 1973a; Lord et al., 1994; Peumans et al., 2001; de Virgilio et al., 2010).

#### Type I RIPs

**FIGURE 1:**
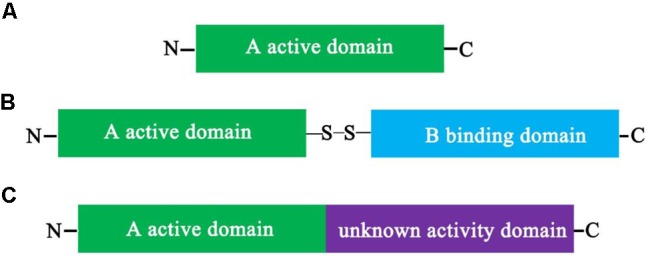
Schematic depiction of the structure of plant ribosome-inactivating proteins (RIPs). Schematic representation of the mature forms of Type I **(A)**, Type II, **(B)** and Type III **(C)** plant RIPs. Comparison between the mature forms of a Type I (RIP) **(A)**, such as α-MMC, composed only of a catalytically active A domain, and that of a Type II RIP **(B)**, such as ricin, in which the active domain is linked to a lectin-binding B domain by a disulfide bond, and that of a Type III RIP **(C)**, such as barley JIP60, in which the active domain is fused to an extra domain with an unknown function. Once the extra domain with the unknown function is removed, the processed active protein is similar in charge and enzymatic activity to type I RIPs.

Type I RIPs are the most widely distributed RIPs, and they are composed of a single polypeptide domain protein of about 30 kDa with *N*-glycosidase activity (Barbieri et al., 1993; Hartley and Lord, 2004a,b; Stirpe, 2004; de Virgilio et al., 2010) (**Figure 1A**). Even though type I RIPs share some of highly conserved active site cleft residues, however, the overall sequence and posttranslational modifications of them are significantly different (Monzingo and Robertus, 1992; Barbieri et al., 1993; Mlsna et al., 1993; Husain et al., 1994). The first type I RIP, pokeweed antiviral protein (PAP), was identified from American pokeweed (Dallal and Irvin, 1978). Subsequently, various type I RIPs have been identified from many different of plant species and a few bacteria species. A large number of isolated type I RIPs are composed of Cucurbitaceae, Euphorbiaceae, and Fabaceae. For example, trichosanthin (TCS), luffin α, luffin β, Mirabilis antiviral protein (MAP), camphorin, barley (*Hordeum vulgare*) translation inhibitor and saporin (from soapwort, *S. officinalis*) are type I RIPs. Meng et al. (2014) combined ion-exchange and gel filtration chromatography methods to isolate two RIPs, α-MMC and an anti-HIV protein (MAP30) from bitter melon. The α-MMC and MAP30 are also type I RIPs with molecular weights of 28.585 and 29.094 kDa, respectively.

#### Low Molecular Weight Type I RIPs

In addition, there are “small type I RIPs” with a low molecular weight compared with the typical type I RIPs. These RIPs are single domain proteins with *N*-glycosidase activity. The N-terminal sequences of these small proteins enrich glutamine and arginine residues. Therefore, they are also considered as arginine/glutamate-rich polypeptides (AGRPs). These AGRPs manifest translation-inhibiting activity. Interestingly, so far, all low molecular weight type I RIPs are identified from Cucurbitaceae family plants. For example, luffacylin, luffangulin, and luffin P1 were isolated from the seeds of *Luffa cylindrical*, and the molecular weights of luffacylin, luffangulin, and luffin P1 are only 7.8, 5.6, and 5.226 kDa, respectively (Parkash et al., 2002a; Wang and Ng, 2002; Li et al., 2003b). Various low molecular weight type I RIPs were isolated and purified from Cucurbitaceae plants, such as α-benincasin and β-benincasin with molecular weights of 12 kDa, which have been isolated and purified from the seeds of *Benincasa hispida* (Ng et al., 2003). In addition, S-trichokirin, trichokirin S1, and trichosanthrip isolated and purified from the seeds of the *Trichosanthes kirilowii* were also low molecular weight type I RIPs. Their molecular weights are only 8, 11.426, and 10.964 kDa, respectively (Tai et al., 2000; Li et al., 2003a; Shu et al., 2009). In addition, Charantin and γ-momorcharin were purified from the seeds of *Momordica charantia* and belong to small type I RIPs, with molecular weights of 9.7 and 11.5 kDa, respectively (Pu et al., 1996; Parkash et al., 2002b).

#### Type II RIPs

Type II RIPs are two-domain polypeptide proteins that include an enzymatically active domain (A domain) of approximately 30 kDa, which is structurally alike to type I RIPs, and a slightly bigger binding domain (B domain) of about 35 kDa that possesses lectin properties (**Figure 1B**). Between the A domain and the B domain is linked by a disulfide bond (Olsnes and Pihl, 1973b; Stirpe et al., 1978). The B-lectin domain possesses sugar-binding properties. For example, it may combine to galactosyl moieties of glycoproteins and/or glycolipids that are discoverd on the surface of eukaryotic cells and mediates retrograde transport of the A-domain by the secretory pathways into the cytosol following by inhibition of protein synthesis (Sandvig and van Deurs, 1994; Steeves et al., 1999). In addition, type I RIPs have a low toxicity due to the lack of the B domain. However, type II RIPs have been broadly divided into two different of groups, nontoxic and toxic based on their cytotoxicity. For example, some type II RIPs, such as modeccin, viscumin, volkensin, abrin, and ricin are highly toxic; in contrast, others type II RIPs, such as iris lectin, cinnamomin, nigrin, and ebulin are nontoxic. The reasons for the differences in toxicities are not completely understood. Type II RIPs were only identified in plants, result in the hypothesis that the connection of RIP and lectin domains happened once in the flowering plant lineage.

#### Type III RIPs

Type III RIPs contain an N-terminal domain which is correlative to the A domain of RIPs and fused to an unknown functional C-terminal domain (**Figure 1C**). Type III RIPs, like barley JIP60 and maize b-32 are synthesized as single-domain proenzymes (Walsh et al., 1991; Chaudhry et al., 1994). RIP1 or b-32 was illustrated as two-domain type I RIP, nevertheless JIP60 was described as a true type III RIP (Nielsen and Boston, 2001; Van Damme et al., 2008). The function of the C-terminal domains (extra domains) of the type III RIP is unknown. When they are removed from the type III RIPs, the active protein is alike in charge and enzymatic activity to type I RIPs (Walsh et al., 1991; Hey et al., 1995; Krawetz and Boston, 2000). For maize, the C-terminal domains (extra domains) are unlikely protective features to prevent self-inactivation of maize ribosomes, because ribosomes from plants are resistant to active RIP and maize proRIP (Hey et al., 1995; Krawetz and Boston, 2000). These RIPs have been characterized only from barley and maize, and they are much less prevalent compared with type I or type II RIPs (Walsh et al., 1991; Bass et al., 1992; Reinbothe et al., 1994).

### Distribution of Ribosome-Inactivating Proteins

Different RIPs have been found in various plant species covering approximately 17 families and in bacteria, fungi and algae (Girbés et al., 2004; Stirpe, 2004). Additionally, RIP-type activity is also reported in some animal tissues (Barbieri et al., 2001). Lots of RIPs were found in a small group of plant species, such as *Caryophyllaceae, Sambucaceae, Euphorbiaceae, Cucurbitaceae, Poaceae, Phytolaccaceae*, and *Rosaceae* (Girbés et al., 2004; Domashevskiy and Goss, 2015; Shang et al., 2016). For example, a type I RIP named TCS was purified from tubers of *T. kirilowii* Maxim. A novel RIP, designated Trichosanthrip, was purified from mature seeds of *T. kirilowii*. Trichosanthrip strongly inhibited cell-free protein synthesis (Shu et al., 2009). Furthermore, RIPs have been proven to locate in various tissues, including leaves, seeds, roots, and tubers (Shu et al., 2009). Some type II RIPs termed Shiga toxins could be produced by bacteria. For example, certain strains of *Escherichia coli* produced type II RIPs Shiga toxin type 1 (Stx1) and Shiga toxin type 2 (Stx2), and their enzymatic activity is similar to their plant analogs (Brown et al., 1980; Sandvig and van Deurs, 2002; Bergan et al., 2012; Melton-Celsa, 2014; Russo et al., 2014). Some studies indicate that RIPs have also been found in different of fungi species, namely, *Volvariella volvacea, Flammulina velutipes, Hypsizigus marmoreus*, and *Lyophyllum shimeji* (Yao et al., 1998; Lam and Ng, 2001a,b; Wang and Ng, 2001). In addition, one RIP has been found in algae (*Laminaria japonica* A) (Liu et al., 2002). In addition, studies suggest that some RIP genes are present in the genome of two mosquitoes (Lapadula et al., 2013, 2017). Overall, RIPs are enzymes, which widely distribute in nature and play significant undefined biological functions.

### The Biological Roles of Ribosome-Inactivating Proteins

#### Role of Ribosome-Inactivating Proteins in Defense against Bacteria

Various RIPs have been shown some unique bioactive properties, including antibacterial (**Table 1**), antifungal and antiviral (Stirpe and Battelli, 2006; Shu et al., 2009). Two type I RIPs isolated from *Mirabilis expansa* roots were active against soilborne bacterial species at microgram levels. In bioassays, the such antibacterial activity was first demonstrated from a plant RIP (Vivanco et al., 1999). The two RIPs ME1 and ME2 exhibited antibacterial activity against *Pseudomonas syringae, Agrobacterium tumefaciens* and *Agrobacterium radiobacter* (Vivanco et al., 1999). A tobacco RIP (TRIP) was isolated and purified from *Nicotiana tabacum* leaves (Sharma et al., 2004). TRIP showed strong *N*-glycosidase activity (Sharma et al., 2004). Purified TRIP showed strong antibacterial activity against *Pseudomonas solanacearum, Erwinia amylovora, Shigella asonei, Salmonella typhimurium*, and *Rhizobium leguminosarum* (Sharma et al., 2004). TRIP at a 50 μg mL^-1^ concentration strongly inhibited *P. solanacearum*. Furthermore, a type I RIP was purified from *Cucurbita moschata*, and it was referred to as *C. moschata* RIP (Barbieri et al., 2006). And it shows antibacterial, superoxide dismutase (SOD) and antifungal activities. It inhibited the growth of two bacterial strains of *E. amylovora* and *P. solanacearum* by 70 and 50%, respectively (Barbieri et al., 2006). Interestingly, α-MMC was successfully expressed in the *E. coli* Rosetta (DE3) and purified by nickel–nitrilotriacetic acid affinity chromatography (Wang et al., 2012). It was active and exhibited antibacterial activity against *Pseudomonas aeruginosa* (Wang et al., 2012). In addition, type I RIP Balsamin isolated from *Momordica balsamina* exhibited broad-spectrum antibacterial activity against *Staphylococcus aureus, Salmonella enterica, Staphylococcus epidermidis*, and *E. coli* (Kaur et al., 2012; Ajji et al., 2016). A type I RIP from *M. balsamina* (MbRIP-1) showed significant antibacterial activity by measuring the radius of suppression from the border of paper disks (Kushwaha et al., 2012). A RIP of *M. jalapa* leaves at concentrations of 0.3–2.5 mg mL^-1^ exhibited antibacterial activity against *Propionibacterium acnes* and *Staphylococcus epidermidis* (Rumiyati et al., 2014). Ricin exhibits the highest activity to mammalian and yeast ribosomes, but presents low activity on *E. coli* and plant ribosomes, however, PAP depurinates ribosomes from yeasts, bacteria, plants, and animals (Barbieri et al., 1993). Furthermore, ricin is active on naked *E. coli* 23S rRNA, whereas is not active against the intact *E. coli* ribosomes (Tang et al., 2001). In a word, RIPs play significant roles in defense against bacteria (**Table 1**).

**Table 1:** The role of different source ribosome-inactivating proteins (RIPs) in defense against bacteria.

RIP	Source		Against bacteria	Reference
	Scientific name	Tissue		
ME1	*Mirabilis expansa*	Roots	*Pseudomonas syringae, Agrobacterium tumefaciens, Agrobacterium radiobacter*	Vivanco et al., 1999
ME2	*Mirabilis expansa*	Roots	*Pseudomonas syringae, Agrobacterium tumefaciens, Agrobacterium radiobacter*	Vivanco et al., 1999
Tobacco RIP (TRIP)	*Nicotiana tabacum*	Leaves	*Pseudomonas solanacearum, Erwinia amylovora, Shigella asonei, Salmonella typhimurium, Rhizobium leguminosarum*	Sharma et al., 2004
*C. moschata* RIP	*Cucurbita moschata*	Sarcocarp	*Erwinia amylovora, Pseudomonas solanacearum*	Barbieri et al., 2006
Alpha-momorcharin	*Momordica charantia*	Seeds	*Pseudomonas aeruginosa*	Wang et al., 2012
MbRIP-1	*Momordica balsamina*	Seeds	*Escherichia coli*	Kushwaha et al., 2012
*M. jalapa* RIP	*Mirabilis jalapa*	Leaves	*Propionibacterium acnes, Staphylococcus epidermidis*	Rumiyati et al., 2014
Balsamin	*Momordica balsamina*	Seeds	*Staphylococcus aureus, Salmonella enterica, Staphylococcus epidermidis, Escherichia coli*	Ajji et al., 2016

#### Role of Ribosome-Inactivating Proteins in Defense against Fungi

Ribosome-inactivating proteins possess extensive interest on account of their potential applications as plant defense agents against viruses and fungi. Luffacylin is a low molecular weight RIP with molecular weights of 7.8 kDa, and the N-terminal sequences of luffacylin enrich glutamate and arginine residues. Furthermore, its chromatographic behavior is alike to charantin. It demonstrates antifungal activity against *Fusarium oxysporum* and *Mycosphaerella arachidicola in vivo* (Parkash et al., 2002a). Two 11 kDa have been proteins are isolated from winter melon (*Benincasa hispida*) seeds (Ng et al., 2003). They exhibited antifungal activity toward *Coprinus comatus* and *Physalospora piricola* (Ng et al., 2003). The isolated TRIP was showed to inhibit several fungi pathogens, including *Trichoderma reesei, Cytospora canker, Fusarium oxysporum*, and *Cochliobolus heterostrophus* (Sharma et al., 2004). The study suggested that TRIP was most active on *T. reesei* among all of the fungi tested. A strong mycelial growth inhibition with 50 μg mL^-1^ TRIP was showed in the time course assay with *T. reesei* (Sharma et al., 2004). *C. moschata* RIP suppressed the growth of *Phytophthora infestans* by more than 60% (Barbieri et al., 2006). A RIP with a molecular weight of approximately 20 kDa was isolated from the seeds of the bottle melon (*Lagenaria siceraria*), and it exhibits *N*-glycosidase and antifungal activities (Wang and Ng, 2000). Two type I RIPs from *M. expansa* roots were found to inhibit both nonpathogenic and pathogenic fungi, *Fusarium* and *Trichoderma* species (Vivanco et al., 1999). Interestingly, the differential sensitivity was found in some cases fungal species from the same genus. For instance, *Pythium irregulare* was found to be sensitive; however, *P. ultimum* was found to be resistant. The type I barley RIP was showed to inhibit the growth of fungal strain of *Trichoderma reesei* on solid media (Roberts and Stewart, 1979). However, barley RIP was minimal to inhibit the growth of *T. reesei* in liquid media but inhibition of growth increased when chitinase was added (Leah et al., 1991). The small RIP luffacylin and Hairy melon RIP also show antifungal activity (Wong et al., 2010). Recently, recombinant DNA technology was utilized to obtain a large number of recombinant proteins (Kushwaha et al., 2012; Zhu et al., 2012). For example, the active and soluble recombinant α-MMC was obtained from the *E. coli* prokaryotic expression system (Wang et al., 2012). Protein activity assay suggested that α-MMC had both DNA-nuclease activity and *N*-glycosidase activity (Wang et al., 2012). Furthermore, the results indicated that the recombinant α-MMC showed a strong mycelial growth inhibition of the filamentous fungi including *Fusarium oxysporum* and *Fusarium solani*, with IC_50_ values of 4.15 and 6.23 μM, respectively (Wang et al., 2012). Further studies suggest that α-MMC isolated from seeds of *Momordica charantia* exhibited antifungal activity (Zhu et al., 2013). The antifungal activity of MbRIP-1 was determined using a radial growth inhibition assay (Kushwaha et al., 2012). The results indicated MbRIP-1 showed significant antifungal activity against *Aspergillus niger* (Kushwaha et al., 2012). Recently, RIPs from *Phytolacca dioica*, dioicin 2 and PD-L4 inhibited the growth of the fungus *Penicillium digitatum* (Iglesias et al., 2016). Novel type I RIPs isolated from oil palm (*Elaeis guineensis*) showed inhibition on *Ganoderma boninense* mycelial growth (Sargolzaei et al., 2016). Therefore, various RIPs exhibited significant antifungal activity (**Table 2**).

**Table 2:** The role of different source RIPs in defense against fungi.

RIP	Source		Against fungi	Reference
	Scientific name	Tissue		
Tobacco RIP (TRIP)	*Nicotiana tabacum*	Leaves	*Trichoderma reesei, Cytospora canker, Fusarium oxysporum, Cochliobolus heterostrophus*	Sharma et al., 2004
*C. moschata* RIP	*Cucurbita moschata*	Sarcocarp	*Phytophthora infestans*	Barbieri et al., 2006
ME1	*Mirabilis expansa*	Roots	*Pythium irregulare, Fusarium oxysporum, Fusarium solani*	Vivanco et al., 1999
ME2	*Mirabilis expansa*	Roots	*Pythium irregulare, Fusarium oxysporum, Fusarium solani*	Vivanco et al., 1999
Alpha-momorcharin	*Momordica charantia*	Seeds	*Bipolaris maydis, Fusarium graminearum, Aspergillus oryzae, Aspergillus niger*	Zhu et al., 2013
MbRIP-1	*Momordica balsamina*	Seeds	*Aspergillus niger, Sclerotinia sclerotiorum*	Kushwaha et al., 2012
Luffacylin	*Luffa cylindrica*	Seeds	*Fusarium oxysporum, Mycosphaerella arachidicola*	Parkash et al., 2002a
Alpha-benincasin	*Benincasa hispida*	Seeds	*Coprinus comatus, Physalospora piricola*	Ng et al., 2003
Barley RIP	*Hordeum vulgare*	Seeds	*Trichoderma reesei*	Roberts and Stewart, 1979
Hispin	*Benincasa hispida var. chieh-qua*	Seeds	*Coprinus comatus, Fusarium oxysporum, Physalospora piricola, Mycosphaerella arachidicola*	Wong et al., 2010
RIP BE27	*Beta vulgaris*	Leaves	*Penicillium digitatum*	Citores et al., 2016
Dioicin 2 and PD-L4	*Phytolacca dioica*	Leaves, seeds	*Penicillium digitatum*	Iglesias et al., 2016
EgRIP-1a and EgRIP-1b	*Elaeis guineensis*	Roots and stems	*Ganoderma boninense*	Sargolzaei et al., 2016

The fungal bioassay offers the advantage of testing the function of a single protein. In addition, some tests can be done with the same concentrations of the target protein equivalent to those found in plant tissues (Dowd et al., 1998). Whereas the employ of a purified protein produces an artificial condition. The actual quantity of RIP would be released from cells during fungal-plant interaction; therefore, it could only be approximated. Transgenic technologies can well solve this question by overexpression of RIPs in transgenic plants. More scientists found that overexpression of RIPs in transgenic plants exhibit enhanced resistance to pathogens attack (**Table 3**). For example, expressing the barley RIP in *Nicotiana tabacum* plants enhanced resistance against the soil-borne pathogen *Rhizoctonia solani* (Logemann et al., 1992; Jach et al., 1995). Co-expressing the barely transgenes β-1,3-glucanase (GLU)/chitinase (CHI) or CHI/RIP in tobacco plants increased significantly resistance against *Rhizoctonia solani* infection (Jach et al., 1995). Expressing the maize proRIP in tobacco plants enhanced resistance against the fungus *R. solani* (Maddaloni et al., 1997). A type I RIP TCS was able to inhibit rice fungus blast disease in transgenic rice (Yuan et al., 2002). A new kind of RIP (curcin 2) isolated from *Jatropha curcas* leaves, can be induced by different kinds of stress (Huang et al., 2008). Expression of curcin 2 in transgenic tobacco plants clearly demonstrated antifungal activity against *R. solani* (Huang et al., 2008). Co-expression of rice basic chitinase (RCH10) and modified maize RIP (MOD1) in rice plants enhanced resistance against *R. solani* (Kim et al., 2003). The barley antifungal gene chitinase and RIP were transformed into Blackgram [*Vigna mungo* (L.) Hepper]. The transgenic plants exhibited improved resistance to Corynespora leaf spot fungal disease (Chopra and Saini, 2014). Recently, expressing the RIP α-MMC confers enhanced resistance to rice blast fungus in rice (Qian et al., 2014). Overexpression of the PhRIP I in transgenic potato plants resulted in antifungal activity against *Botrytis cinerea* and *R. solani* (Gonzales-Salazar et al., 2017). Therefore, further work is necessary to screen the high germination rate and strong resistance to rice blast fungus in transgenic rice for applications in agriculture. From the standpoint of agricultural biotechnology applications, ectopic expression studies with transgenic plants is valuable. However, discovering that RIPs can reduce susceptibility to damage by different of pathogens does not eliminate more important roles for these proteins. To discover such roles, a comparative analysis of plants involving the alteration of RIP function by mutation, insertional inactivation, or co-suppression will be carried out.

**Table 3:** Transgenic plant species expressing RIPs show enhanced resistance to fungi infection.

RIP	Transgenic plant species	Against fungi	Reference
	expressing RIPs		
Barley RIP	*Nicotiana tabacum*	*Rhizoctonia solani*	Logemann et al., 1992; Jach et al., 1995
Barley RIP	*Vigna mungo*	*Corynespora leaf spot fungal disease*	Chopra and Saini, 2014
Maize proRIP	*Nicotiana tabacum*	*Rhizoctonia solani*	Maddaloni et al., 1997
Trichosanthin (TSC)	*Oryza sativa*	*Rice fungus blast disease*	Yuan et al., 2002
Curcin 2	*Nicotiana tabacum*	*Rhizoctonia solani*	Huang et al., 2008
Modified maize RIP (MOD1)	*Oryza sativa*	*Rhizoctonia solani*	Kim et al., 2003
α-MMC	*Oryza sativa*	*Rice blast fungus*	Qian et al., 2014
PhRIP I	*Solanum tuberosum*	*Botrytis cinerea, Rhizoctonia solani*	Gonzales-Salazar et al., 2017

It is believed that the antifungal proteins, namely, RIP, thaumatin-like proteins, chitinases, proteinase inhibitors, endoproteinases, plant defensins, glucanases, peroxidases, and immunophilin, utilize protective activity against fungal and bacterial invasions (Park et al., 2009). Antifungal proteins can disrupt the cell wall structure or function and suppress the synthesis of the fungal cell wall (Morais et al., 2010). Some antifungal proteins can interact with the potential fungal intracellular targets and plasma membrane, leading to changes in the cell death or membrane potential (Morais et al., 2010). Wang et al. (2012) investigated the antifungal activity and mechanism of α-MMC toward *F. solani*. The results of the DAPI and PI uptakes assay indicated that the apoptotic responses can be induced and the permeability of fungal membranes can be changed by α-MMC (Wang et al., 2012). The ultrastructural changes, such as cells with irregular budding sites, other aspect in the cytoplasm, the loss of integrity and rigidity of cell walls suggested that the antifungal mechanism of α-MMC was a complex process (Wang et al., 2012). Recently, the antifungal activity of the RIP BE27 isolated from sugar beet was investigated against the green mold *Penicillium digitatum* (Citores et al., 2016). They found that RIP BE27 can inhibit the growth of the fungus *Penicillium digitatum* because that it can enter the fungal cytoplasm and kill fungal cells (Citores et al., 2016).

#### Role of Ribosome-Inactivating Proteins in Defense against Plant Viruses

The existence of antiviral activities of RIPs are known for about 75 years. RIPs emerged as potent antiviral agents against lots of animal, plant and human viruses. This review summarizes work related to the role of RIPs in defense against plant viruses (**Tables 4, 5**). PAP is a RIP that is purified from the extracts of plant leaves of pokeweed (Obrig et al., 1973). It was first shown to reduce the infectivity of TMV by inhibiting protein synthesis (Duggar and Armstrong, 1925; Dallal and Irvin, 1978). Further studies suggested that the exogenous application of PAP appears to enhance the systemic resistance of *N. benthamiana* to TMV infection (Zhu et al., 2016). Application of PAP I also causes concentration-dependent inhibition of zucchini yellow mosaic virus (ZYMV) infection on squash plants (Sipahioğlu et al., 2017). Dianthins isolated from the leaves of *Dianthus caryophyllus* mixed with TMV strongly decreased the amount of local lesions on plant leaves of *Nicotiana glutinosa* (Stirpe et al., 1981). The MAP isolated from *M. jalapa* showed highly potent activity against the mechanical transmission of viruses, including cucumber mosaic virus (CMV), TMV, potato Y virus, cucumber green mottle mosaic virus and turnip mosaic potyvirus (TuMV) (Kubo et al., 1990). The infection of artichoke mottled crinkle virus (AMCV) can be inhibited by the new single-chain RIPs from the seeds of *Basella rubra* and from the leaves of *Bougainvillea spectabilis* (Bolognesi et al., 1997). In addition, the translation progress of pokeweed mosaic virus (PMV) and brome mosaic virus (BMV) can be inhibited by an antiviral protein isolated from plant leaves of *Celosia cristata* (Baranwal et al., 2002). A 27 kDa RIP purified from *Amaranthus tricolor* leaves exhibited antiviral activity against the *sunn-hemp rosette virus* (SRV) (Roy et al., 2006). Choudhary N. et al. (2008) purified a recombinant RIP named BBAP1, which showed *N*-glycosidase activity, and emerged strong resistance against TMV. A RIP from *Bougainvillea xbuttiana* leaves was also successfully expressed in *E. coli*. And it demonstrated a high level of resistance against SRV (Choudhary N.L. et al., 2008). In addition, the activity of RIPs against capped and uncapped viral RNAs were examined. Vivanco and Tumer (2003) found that PAP, ME1, and saporin can depurinate the capped TMV and BMV RNAs. However, the uncapped luciferase RNA are not depurinated by them. Taken together, various RIPs purified from different plant species exhibited significant antiviral activity (**Table 4**).

**Table 4:** The role of different source RIPs in defense against viruses.

RIP	Source		Against viruses	Reference
	Scientific name	Tissue		
Alpha-momorcharin	*Momordica charantia*	Seeds	*Chilli veinal mottle virus, Cucumber mosaic virus, Tobacco mosaic virus, Turnip mosaic potyvirus*	Zhu et al., 2013; Yang et al., 2016
PAP	*Phytolacca americana*	Leaves	*Tobacco mosaic virus*	Dallal and Irvin, 1978; Zhu et al., 2016
New single-chain RIPs	*Basella rubra*	Seeds	*Artichoke mottled crinkle virus*	Bolognesi et al., 1997
New single-chain RIPs	*Bougainvillea spectabilis*	Leaves	*Artichoke mottled crinkle virus*	Bolognesi et al., 1997
CCP 25	*Celosia cristata*	Leaves	*Brome mosaic virus, Pokeweed mosaic virus*	Baranwal et al., 2002
27-kDa RIP	*Amaranthus tricolor*	Leaves	*Sunn-hemp rosette virus*	Roy et al., 2006
RIP	*Bougainvillea xbuttiana*	Leaves	*Sunn-hemp rosette virus*	Choudhary N.L. et al., 2008
ME1	*Mirabilis expansa*	Roots	*Tobacco mosaic virus, Brome mosaic virus*	Vivanco and Tumer, 2003
Clerodendrum aculeatum-systemic resistance inducing (CA-SRI) protein	*Clerodendrum aculeatum*	Leaves	*Tobacco mosaic virus, Sunn-hemp rosette virus*	Verma et al., 1996; Kumar et al., 1997
CAP-34	*Clerodendrum aculeatum*	Leaves	*Papaya ringspot virus*	Srivastava et al., 2009
CIP-29	*Clerodendrum inerme*	Leaves	*Tobacco mosaic virus*	Prasad et al., 1995; Olivieri et al., 1996
BDP-30	*Boerhavia diffusa*	Roots	*Tobacco mosaic virus*	Srivastava et al., 2015
PAP I	*Phytolacca americana*	Leaves	*Zucchini yellow mosaic virus*	Sipahioğlu et al., 2017

**Table 5:** Transgenic plant species expressing RIPs show enhanced resistance to virus infection.

RIP	Transgenic plant species expressing RIPs	Against viruses	Reference
Pokeweed antiviral protein	*Tobacco (Nicotiana tabacum and Nicotiana benthamiana)*	*Cucumber mosaic virus, Potato virus Y, Potato virus X*	Lodge et al., 1993; Zoubenko et al., 2000
Pokeweed antiviral protein	*Solanum tuberosum*	*Cucumber mosaic virus, Potato virus Y, Potato virus X*	Lodge et al., 1993
Phytolacca insularis antiviral protein	*Solanum tuberosum*	*Potato virus Y, Potato virus X, Potato leafroll virus*	Moon et al., 1997
Trichosanthin	*Nicotiana tabacum*	*Turnip mosaic virus, Cucumber mosaic virus, Tobacco mosaic virus*	Lam et al., 1996; Krishna et al., 2002
Cassin	*Tobacco (Nicotiana tabacum)*	*Tobacco mosaic virus*	Ruan et al., 2007
Curcin 2	*Tobacco (Nicotiana tabacum)*	*Tobacco mosaic virus*	Huang et al., 2008
Type-2 RIP SNA-I’	*Tobacco (Nicotiana tabacum)*	*Tobacco mosaic virus*	Chen et al., 2002

Biotechnological methods have been used to show a significant role of RIPs in plant defense against viral infections (**Table 5**). Different RIPs, including PAP and TCS have been successfully overexpressed in different of transgenic plant species, leading to improved resistance against multiple plant viruses (Lodge et al., 1993; Taylor et al., 1994; Lam et al., 1996; Moon et al., 1997). A gene encoding a RIP, named *P. insularis* antiviral protein 2 (PIP2) was isolated from *P. insularis* (Song et al., 2000). They also found that the expression of the *PIP2* gene is enhanced in leaves after treatment with phytohormones, such as abscisic acid (ABA) and jasmonic acid (JA). Furthermore, the PIP2 protein exhibited antiviral activity against TMV. The results indicate that the antiviral activity of PAP was not due to the depurination of host ribosomes. A mutant PAP, PAPn, did not bind ribosomes efficiently. PAPn did not depurinate ribosomes and was non-toxic when expressed in transgenic tobacco plants (Zoubenko et al., 2000). However, overexpression of PAPn in tobacco plants increased resistance to the potato virus X (PVX) infection (Zoubenko et al., 2000). They presented evidence that a novel SA-independent, stress-associated signal transduction pathway activated in PAPn-expressing plants play a significant role in pathogen resistance against virus infection (Zoubenko et al., 2000). Transgenic plants expressing the C-terminal deletion mutant PAP were resistant to PVX infection. However, the depurination of rRNA was not found in these plants (Tumer et al., 1997). In addition, Hudak et al. (2000) showed that PAP inhibited the translation of BMV and PVX RNAs without depurinating ribosomes. Overall, overexpression of PAP and non-toxic PAP mutants increased broad-spectrum resistance to viral infection in transgenic plants. Di and Tumer (2015) have summarized the mechanism of PAP-induced disease resistance. Ruan et al. (2007) suggested that overexpressing Cassin, a new gene of RIP isolated from *Cassia occidentalis* in tobacco plants exhibited different levels of resistance to TMV. The expression of curcin 2 in transgenic tobacco plants clearly demonstrated antiviral activity against TMV (Huang et al., 2008). The expression of a type I RIP TCS enhanced resistance to CMV and TMV infection (Krishna et al., 2002). The symptoms of systemic infection were weakened and delayed in the transgenic plants overexpressing TCS. Most of the evidence suggest that overexpression of the type I RIPs increases the plant’s resistance against various plant viruses in transgenic plants. In order to confirm whether overexpression of type II RIPs also enhance the plant’s resistance against viruses infection, Chen et al. (2002) transformed the *Sambucus nigra* type II RIP SNA-I’ into tobacco plants. Overexpression of SNA-I’ strongly enhanced the plant’s resistance against TMV infection in transgenic tobacco plants (Chen et al., 2002). These results suggest that type II RIPs are also involved in plant defense response against viral infection.

A cDNA clone, encoding RIP was isolated from the post-flowering stage of *C. cristata* leaves (Begam et al., 2006). The recombinant protein was successfully expressed in *E. coli* and showed antiviral activity against TMV and SRV (Begam et al., 2006). Similarly, a full-length cDNA encoding RIP from the leaves of *B. xbuttiana* was isolated, and this 35.5 kDa RIP was also successfully expressed in *E. coli* (Choudhary N.L. et al., 2008). The 35.5-kDa RIP can inhibit the local lesion formation of SRV infecting *Cyamopsis tetragonoloba* leaves, which indicates that it exhibited antiviral activity toward SRV (Choudhary N.L. et al., 2008). In addition, the antiviral potential of RIPs for plant viruses and its potential function in systemic resistance were also studied. The *Clerodendrum aculeatum*-systemic resistance inducing (CA-SRI) protein, which has RIP activity, plays a key role in inducing strong systemic resistance in susceptible plants against various plant viruses (Verma et al., 1996; Kumar et al., 1997). Further studies indicate that CAP-34, a protein from *C. aculeatum* induced systemic antiviral resistance against the papaya ringspot virus (PRSV) infection in *Carica papaya* (Srivastava et al., 2009). Likewise, the CIP-29 protein isolated from *Clerodendrum inerme* exhibited ribosome-inactivating properties. Compared with other known RIPs, CIP-29 can induce systemic resistance against virus infection in susceptible plants (Prasad et al., 1995; Olivieri et al., 1996). Further study suggest that application of CIP-29 in *C. tetragonoloba* plants can induce two virus inhibitory proteins, named CT-VIA-32 and CT-VIA-62 (Prasad et al., 2014). These two virus inhibitory proteins can resist virus infection. Systemic resistance in *N. glutinosa* and *Cyamopsis tetragonoloba* was induced by an antiviral protein from *B. xbuttiana* leaves against TMV and SRV (Narwal et al., 2001). Iglesias et al. (2005) found that sugar beet RIPs named beetins were systemically induced by AMCV infection. BDP-30, a glycoprotein isolated from *Boerhavia diffusa* displays homology with RIPs. It also induced systemic resistance in tobacco against TMV (Srivastava et al., 2015). Zhu et al. (2013) demonstrated that α-MMC had broad-spectrum antiviral activity against phytopathogenic viruses, including ChiVMV, CMV, TMV, and TuMV. The results demonstrated that α-MMC inhibited the accumulation of viral RNA and protein. Furthermore, application of α-MMC can increase the expression of *NPR1, PR1*, and *PR2* in tobacco plants, which strongly indicates that the plant systemic resistance can be activated by α-MMC against multiple viruses (Zhu et al., 2013). Furthermore, application of α-MMC in *M. charantia* led to a significant increase of JA indicated that the anti-viral activities of α-MMC in *M. charantia* may be accomplished through the JA related signaling pathway (Yang et al., 2016). Therefore, it may be a candidate for the development of virus resistant crop plants, for instance cotton, tobacco, rice, tea, and clover. Although RIPs exhibited antiviral activity against various plant viruses, viruses also have evolved a viral strategy to evade host cell defenses and possible anti-cytotoxic activity against RIPs. The interactions between the turnip mosaic virus genome linked protein (VPg) and PAP was investigated (Domashevskiy et al., 2012). PAP interacts strongly with VPg in a mixed type competition. These findings correlate with the inhibition of PAP enzymatic activity by VPg in wheat germ lysate. Furthermore, depurination inhibition by VPg also indicates the use of VPg against the cytotoxic activity of RIPs and the inhibition of their biological potency (Domashevskiy et al., 2012).

A large number of type I and type II RIPs have been shown to have antiviral effect; however, the antiviral mechanism of RIPs is still not completely clear (Wang and Tumer, 2000). The antiviral effect of RIPs is supposed on the basis of their enzymatic activity and selective compartmentalization (Roberts and Lord, 1981; Ready et al., 1986; Carzaniga et al., 1994; Tarantini et al., 2010). RIPs may work on ribosomes of infected plant cells, thereby inhibiting the synthesis of viral protein (Parikh and Tumer, 2004). When plants are infected by plant viruses, RIPs can be released rapidly from their intracellular compartments. Therefore, viral replication can be prevented at an early stage by inhibiting the cell protein synthesis and leading to autonomous cell death (Wang and Tumer, 2000; Nielsen and Boston, 2001). However this antiviral mechanism of RIPs may have a direct interaction of RIP with nucleic acid of viruses. Vandenbussche et al. (2004) considered that RIP may have a direct effect on the nucleic acid of viruses. PAP-I, PAP-II, and PAP-III can cause a concentration-dependent depurination of genomic TMV RNA (Chen et al., 1993), BMV (Picard et al., 2005), and tobacco etch virus (TEV) RNA (Domashevskiy et al., 2012). The antiviral mechanism of RIPs may change among different RIPs and different viruses.

#### Role of Ribosome-Inactivating Proteins in Defense against Insects

RIPs can enhance plant resistance to insect pests (Stirpe, 2013) (**Table 6**). Lots of studies have suggested that RIPs possess the insecticidal activity upon different insects such as Lepidoptera (Dowd et al., 1998, 2003, 2006; Zhou et al., 2000; Wei et al., 2004), Coleoptera (Gatehouse et al., 1990; Kumar et al., 1993) and Diptera (Shahidi-Noghabi et al., 2008). The artificial diets experiments were used to study the insecticidal activity of RIPs. For example, an artificial diet supplemented with different concentrations of a type-II RIP from *Sambucus nigra*, decreased the fecundity and survival of *Acyrthosiphon pisum* (Shahidi-Noghabi et al., 2008). Furthermore, the feeding of *Myzus nicotianae* with leaves from transgenic tobacco plants overexpressing SNA-I retarded the development and decreased the fecundity and adult survival (Shahidi-Noghabi et al., 2008). In addition, an artificial diet supplemented with different type-I RIPs reduced the fecundity and survival of *Anticarsia gemmatalis* Hübner and *Spodoptera frugiperda* (Bertholdo-Vargas et al., 2009). Recent studies have suggested that type-I and type-II RIPs from apple (*Malus domestica* Borkh) have strong aphicidal activity (Hamshou et al., 2016). The feeding of pea aphids (*Myzus nicotianae* Blackman) on an artificial diet supplemented with the purified recombinant proteins for type-I RIPs and type-II RIPs from apple reduced the nymphal survival of aphid (Hamshou et al., 2016). The studies also indicated that the feeding of green peach aphids (*Myzus persicae* Sulzer) on leaves of different transgenic tobacco plants lines overexpressing type-I or type-II RIPs significantly reduced nymphal survival of this important hemipteran pest. In addition, Overexpression of a maize RIP in tobacco plants enhanced resistance against *Helicoverpa zea* (Dowd et al., 2003). Furthermore, Maize leaves expressing higher levels of maize ribosome-inactivating protein (MRIP) and wheat germ agglutinin (WGA) were more resistant to feeding by *Spodoptera frugiperda* and corn earworms (*Helicoverpa zea*) (Dowd et al., 2012). Overexpression of type 1 or type 2 RIPs from apple in tobacco plants enhanced resistance against agronomically important insect pest, *Spodoptera exigua* (Hamshou et al., 2017). The mechanism of insecticidal activity of RIPs is still not completely clear. Several studies suggested that apoptosis can be induced by RIPs (Narayanan et al., 2005; Sikriwal and Batra, 2010; Das et al., 2012). The feeding of *A. pisum* on an artificial diet supplemented with SNA-I induced apoptosis in the midgut of *A. pisum* through the activation of caspase-3 (Shahidi-Noghabi et al., 2010).

**Table 6:** The Role of different source RIPs in defense against insects.

RIP	Source		Against insects	Administration	Reference
	Scientific name	Tissue			
Ricin	*Ricinus communis*	Seeds	*Callosobruchus maculatus, Abies grandis, Bombyx mori*	Artificial diet, air-dried onto leaf	Gatehouse et al., 1990; Wei et al., 2004
SNA-I	*Sambucus nigra* L.	Bark	*Acyrthosiphon pisum, Myzus nicotianae*	Artificial diet, transgenic tobacco	Shahidi-Noghabi et al., 2008
Lectin	*Branthis hyemdis*	Bulbs	*Diabrotica undecimpunctata*	Artificial diet	Kumar et al., 1993
*Cinnamomin*	*Cinnamomum camphora*	Seeds	*Helicoverpa armigera, Culex pipiens Pallens, Bombyx mori*	Artificial diet, oral feeding	Zhou et al., 2000; Wei et al., 2004
Maize RIP	*Zea mays*	Seeds	*Helicoverpa zea, Lasioderma serricorne*	Transgenic tobacco	Dowd et al., 2003, 2006
Saporin	*Saponaria officinalis*	Seeds	*Anticarsia gemmatalis, Spodoptera frugiperda*	Air-dried onto leaf	Bertholdo-Vargas et al., 2009
PAP-S	*Phytolacca americana*	Leaves	*Anticarsia gemmatalis, Spodoptera frugiperda*	Air-dried onto leaf	Bertholdo-Vargas et al., 2009
Lychnin	*Lychnis chalcedonica*	Seeds	*Anticarsia gemmatalis, Spodoptera frugiperda*	Air-dried onto leaf	Bertholdo-Vargas et al., 2009
Gelonin	*Gelonium multiflorum*	Seeds	*Anticarsia gemmatalis, Spodoptera frugiperda*	Air-dried onto leaf	Bertholdo-Vargas et al., 2009
Momordin	*Momordica charantia*	Seeds	*Anticarsia gemmatalis, Spodoptera frugiperda*	Air-dried onto leaf	Bertholdo-Vargas et al., 2009
Type-1 RIP	*Malus domestica*	Leaves	*Acyrthosiphon pisum, Myzus persicae, Spodoptera exigua*	Artificial diet, transgenic tobacco	Hamshou et al., 2016, 2017
Type-2 RIP	*Malus domestica*	Leaves	*Acyrthosiphon pisum, Myzus persicae, Spodoptera exigua*	Artificial diet, transgenic tobacco	Hamshou et al., 2016, 2017
MRIP	*Zea mays*	Seeds	*Spodoptera frugiperda, Helicoverpa zea*	Artificial diet, transgenic maize	Dowd et al., 2012

### Conclusion and Perspectives

**FIGURE 2:**
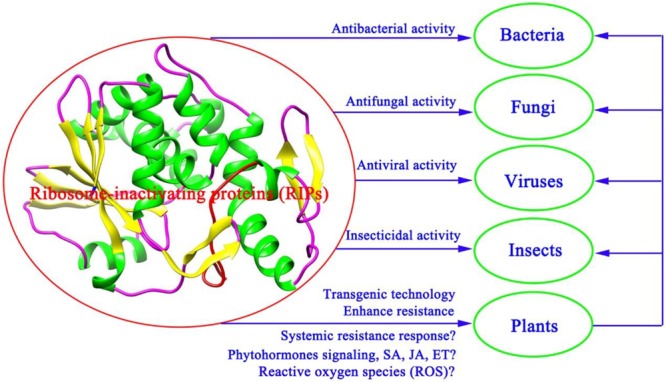
A proposed model for the role of RIPs in defense against pathogens and insects.

As shown in **Figure 2**, the proposed model for the role of RIPs in defense against pathogens showed that various RIPs have shown unique bioactive properties, including antibacterial, antifungal, antiviral, and insecticidal activity (Stirpe and Battelli, 2006; Shu et al., 2009; Akkouh et al., 2015). A large number of studies suggest that the expression of RIPs could be activated by some factors, such as viral infection, phytohormones, senescence, development, and environmental stress (Reinbothe et al., 1994; Girbés et al., 1996; Stirpe et al., 1996; Rippmann et al., 1997; Wang et al., 2016). Expressing α-MMC by transgenic technologies in rice confers enhanced resistance to rice blast fungus (Qian et al., 2014). Future research employing transgenic technology approaches to study the mechanisms of RIPs will undoubtedly lead to a better comprehending of the effect of plant RIPs in defense against pathogens and insects (**Figure 2**). A deeper insight into antiviral mechanisms should also be carried out (**Figure 2**). Phytohormones salicylic acid (SA), JA, and ethylene (ET) have been proved to participate in the systemic resistance responses of plants against pathogen infections (van Wees et al., 2000). These phytohormones signaling pathways could impact each other via a complex signaling network (Koornneef and Pieterse, 2008). Therefore, how RIPs participate in the systemic resistance responses of plants or whether RIPs can regulate phytohormone signaling pathways (SA, JA, and ET) against pathogen infections needs further investigation (**Figure 2**). Previous studies suggest that PAP may enhance plant systemic resistance against TMV infection by regulation the reactive oxygen species (ROS) levels (Zhu et al., 2016). As shown in **Figure 2**, the proposed model for the role of RIPs in defense against pathogens and insects indicated that discovering additional crosstalk mechanisms between RIPs and phytohormones or ROS against pathogen and insect infections will be a significant subject in the field of biotic stress study.

## Author Contributions

FZ wrote the manuscript. Y-KZ, Z-LJ, and X-RC collected the data and the references. All authors read and approved the final manuscript.

## Conflict of Interest Statement

The authors declare that the research was conducted in the absence of any commercial or financial relationships that could be construed as a potential conflict of interest.
